# Motor mapping of the hand muscles using peripheral innervation‐based navigated transcranial magnetic stimulation to identify functional reorganization of primary motor regions in malignant tumors

**DOI:** 10.1002/hbm.26642

**Published:** 2024-03-04

**Authors:** Haosu Zhang, Wei Zhang, Ann‐Katrin Ohlerth, Maximilian Schwendner, Axel Schröder, Bernhard Meyer, Sandro M. Krieg, Sebastian Ille

**Affiliations:** ^1^ Department of Neurosurgery Technical University of Munich, School of Medicine Munich Germany; ^2^ Department of Neurosurgery Heidelberg University Hospital Heidelberg Germany; ^3^ Center for Language and Cognition Groningen University of Groningen Groningen Netherlands; ^4^ TUM‐Neuroimaging Center Technical University of Munich, School of Medicine Munich Germany

**Keywords:** glioblastoma, motor, plasticity, reorganization

## Abstract

Tumor‐related motor reorganization remains unclear. Navigated transcranial magnetic stimulation (nTMS) can investigate plasticity non‐invasively. nTMS‐induced motor‐evoked potentials (MEPs) of different muscles are commonly used to measure the center of gravity (CoG), the location with the highest density of corticospinal neurons in the precentral gyrus. We hypothesized that a peripheral innervation‐based MEP analysis could outline the tumor‐induced motor reorganization with a higher clinical and oncological relevance. Then, 21 patients harboring tumors inside the left corticospinal tract (CST) or precentral gyrus were enrolled in group one (G1), and 24 patients with tumors outside the left CST or precentral gyrus were enrolled in Group 2 (G2). Median‐ and ulnar‐nerve‐based MEP analysis combined with diffusion tensor imaging fiber tracking was used to explore motor function distribution. There was no significant difference in CoGs or size of motor regions and underlying tracts between G1 and G2. However, G1 involved a sparser distribution of motor regions and more motor‐positive sites in the supramarginal gyrus—tumors inside motor areas induced motor reorganization. We propose an “anchor‐and‐ship theory” hypothesis for this process of motor reorganization: motor CoGs are stably located in the cortical projection area of the CST, like a seated anchor, as the core area for motor output. Primary motor regions can relocate to nearby gyri via synaptic plasticity and association fibers, like a ship moving around its anchor. This principle can anticipate functional reorganization and be used as a neuro‐oncological tool for local therapy, such as radiotherapy or surgery.

## Graphical abstract

We propose an “anchor‐and‐ship theory” for motor reorganization: centers of gravity are stably located in the cortical projection area of the corticospinal tract (seated anchor), as the core area for motor output. Primary motor regions can relocate to nearby gyri via synaptic plasticity, like a ship moving around its anchor.

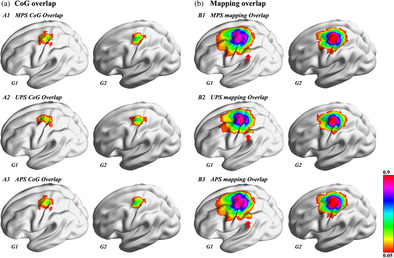

**Abbreviations**
All‐FA: APS‐related average FA
All‐volume: APS‐related CST volume
APS: all positive stimuli
AR: aspect ratio
CoG: center of gravity
CST: corticospinal tract
DTI: diffusion tensor imaging
FA: fractional anisotropy
G1: group of patients with tumors inside the left CST or precentral gyrus
G2: group of patients with tumors outside the left CST or precentral gyrus
Median‐FA: MPS‐related average FA
Median‐volume: MPS‐related CST volume
MEP: motor‐evoked potential
MPS: median‐related positive stimuli
MS: mapping sizes
nTMS: navigated transcranial magnetic stimulation
PS: positive site
rMT: resting motor threshold
Ulnar‐FA: UPS‐related average FA
Ulnar‐volume: UPS‐related CST volume
UPS: ulnar nerve‐related positive stimuli

## 1 INTRODUCTION

The traditional understanding of the functional organization in the brain suggests that primary motor loci are situated similarly across individuals, typically within regions statically like the precentral gyrus (Duffau, 2005). However, recent imaging studies have revealed interindividual variability in the cerebral functional organization, even among healthy volunteers, challenging this notion and highlighting plasticity (Sollmann et al., 2021; Tzourio‐Mazoyer et al., 2004). This reorganization is thought to be the potential mechanism in the case of low‐grade gliomas slowly infiltrating the so‐called primary functional regions but without inducing detectable neurological deficits (Duffau, 2005).

Malignant tumors, such as high‐grade gliomas and metastases, show shorter courses of disease compared to low‐grade gliomas resulting in serious functional deficits due to their rapid and invasive development, which seemingly precludes the effects of functional plasticity. Nevertheless, reorganizations of motor functions at cortical levels were described in a study by Krings et al. enrolling glioma patients (Atlas et al., 1996; Krings et al., 2002). However, previous studies mostly failed to elucidate the exact mechanisms and the extent of functional deficits related to plasticity (Bulubas et al., 2018; Jones, 2017). Therefore, a comprehensive analysis of the motor function in patients with malignant tumors inside or outside primary motor regions is imperative to discern variations in motor plasticity as a response to functional impairments caused by malignant tumors.

Furthermore, the identification of brain areas capable of compensating for the function of the tumor lesion site, thus enabling the protection of brain function during surgery to achieve maximum tumor resection, is warranted.

Navigated transcranial magnetic stimulation (nTMS) mapping, which induces motor‐evoked potentials (MEPs), is routinely utilized in clinical practice to localize motor‐related cortical regions (Conway et al., 2017). The method of nTMS has demonstrated a high degree of spatial specificity and sensitivity, closely compliant with motor mapping by applying the gold standard of intraoperative direct cortical stimulation (Haddad et al., 2020; Jeltema et al., 2020; Sollmann et al., 2021; Tarapore et al., 2012).

Recent literature suggests that certain aspects of nTMS need to be further investigated. First, the analysis of nTMS motor mapping results often involved the center of gravity (CoG) of motor regions through coordinates of positive stimulation targets and their corresponding MEPs (Ardila et al., 2016; Conway et al., 2017). Classen et al. identified nTMS‐derived CoG projections on the posterior lip of the precentral gyrus, a location corresponding to the densest concentration of corticospinal neurons (Classen et al., 1998). Second, cortical motor regions can further serve as seeds in diffusion tensor imaging (DTI) fiber tracking to identify subcortical structures integral to motor function, among which the corticospinal tract (CST) is the particularly important structure influencing the motor control (Conti et al., 2014; Yousry et al., 1997), and its projection on the cortex is considered to influence fine motor function, mainly (Lacroix et al., 2004). DTI fiber tracking has been employed for assessing the risk of motor deficits preoperatively and during tumor resection (Lam et al., 2019; Vitikainen et al., 2013). Third, previous studies collected MEPs from the abductor pollicis brevis muscle, abductor digiti minimi muscle, and flexor carpi radialis muscle to broadly define motor regions. It should be noticed that different peripheral nerves innervate those muscles: the median nerve for the abductor pollicis brevis muscle and flexor carpi radialis muscle and the ulnar nerve for the abductor digiti minimi muscle. Analyzing these nerve responses separately offers a more detailed view of the cortical regions for controlling peripheral nerves. Since the median and ulnar nerves are essential to maintaining hand motor function, mapping cortical distributions for these two nerves can improve understanding of motor plasticity in tumor patients. Therefore, cortical regions and subcortical CST projections related to the median and ulnar nerve are investigated in our study.

The current study examines a cohort of patients with malignant tumors inside and outside primary motor regions undergoing nTMS to localize their preoperative cortical and subcortical distributions of motor function.

The aim of this study was to evaluate the impact of the tumor location on the cortical reorganization of motor function, including the localization of the CoG and the underlying subcortical structures, such as the CST, with motor function classified by the functional distribution in peripheral nerves of the upper extremities.

## 2 MATERIALS AND METHODS

### 2.1 Ethics approval

The current study was approved and supervised by our local ethics committee and is by the Declaration of Helsinki (registration number: 222/14, 192/18). All patients were given detailed study information and written consent before preoperative nTMS motor mapping.

### 2.2 Patients' enrollment

The enrolment standards were as follows: (1) right‐handedness according to Edinburgh Handedness Inventory scales (Oldfield, 1971); (2) age >18 years; (3) primary left‐sided high‐grade glioma or metastasis; (4) no previous brain surgery before the current enrollment. The exclusion standards were: (1) any contraindication to MRI scanning or nTMS mapping and (2) lesions in both hemispheres.

**FIGURE 1:**
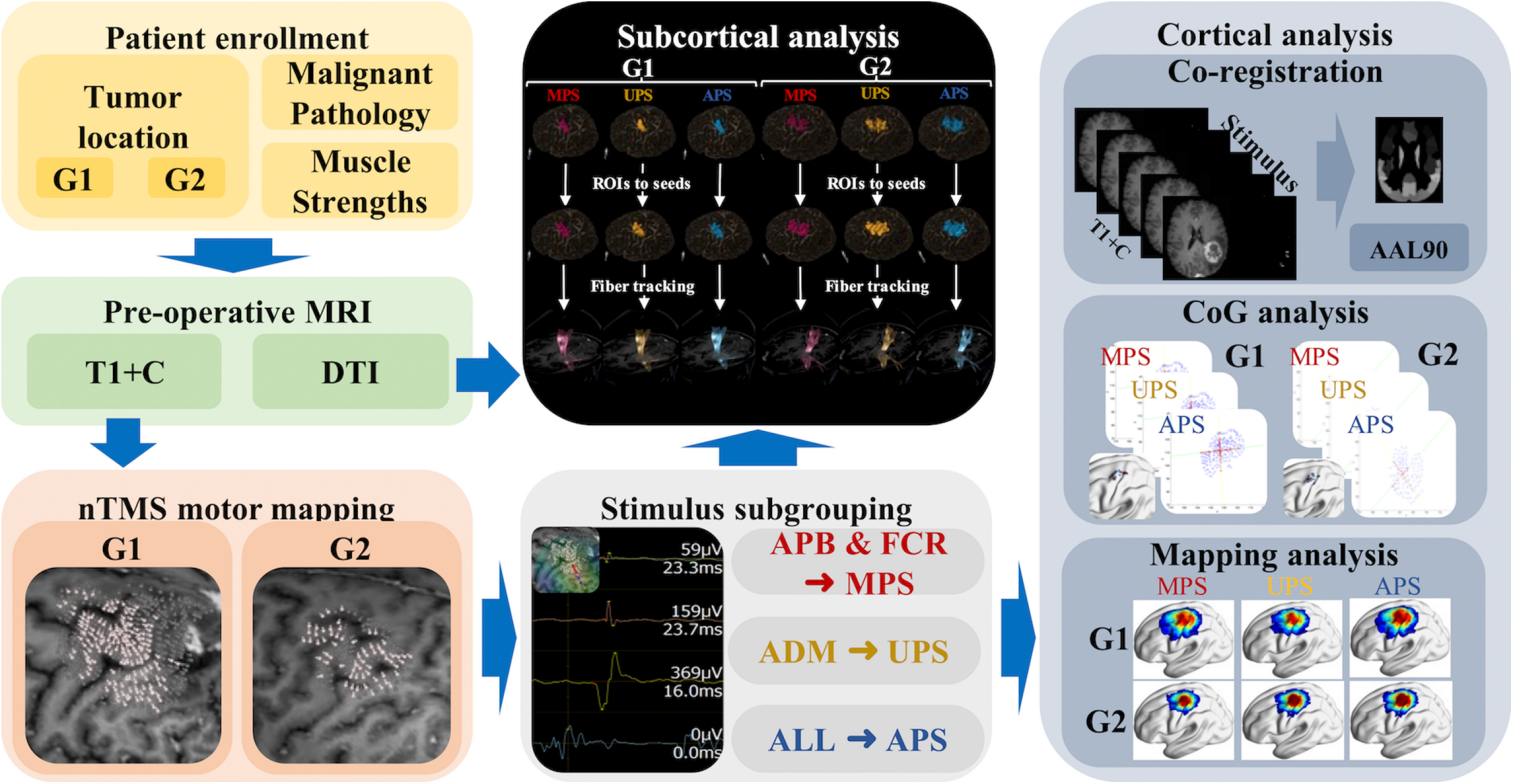
Workflow of the study. This figure presents the workflow of the current study. It consists of a cortical and subcortical part. In the yellow box, group 1 (patients with tumors inside the left corticospinal tract [CST] or precentral gyrus, G1) and group 2 (patients with tumors outside the left CST or precentral gyrus, G2) are defined by whether tumors are within or outside primary motor regions. In the green box, their T1 images with contrast (T1 + C) are applied for Navigated transcranial magnetic stimulation (nTMS) motor mapping, and the diffusion tensor imaging (DTI) files are imported to the Brainlab Element system for subcortical analysis (black box). According to different innervation, motor‐evoked potentials (MEPs) from other muscles are categorized into three groups. MEPs >50 mv are considered positive stimuli (PS). As shown in the gray box, PSs from the left abductor pollicis brevis muscle (APB) and flexor carpi radialis muscle (FCR) are grouped to median nerve‐related PSs (MPS), PSs from abductor digiti minimi muscle (ADM) are categorized as ulnar nerve‐related PSs (UPS), and all PSs are summarized as All PSs (APS). The cortical analysis of the center of gravity and mapping regions is depicted in the blue box. For the subcortical analysis in the black box, the areas of interest, respectively, from the MPS, UPS, and APS, are applied for the seed‐based fiber tracking analysis.

According to tumor location on MRI scans, 21 patients (2016–2020) with tumor entities inside the left CST or precentral gyrus were grouped as group one (G1), and 24 patients (2016–2020) with tumor entities located in brain regions except the left CST or precentral gyrus and with intact motor function were grouped as Group 2 (G2) (Figure 1). Demographic data are shown in Supplementary Table 1.

### 2.3 Data collection and analysis

The workflow is shown in Figure 1. Pathological diagnoses and pre‐ and postoperative muscle strengths were collected. MRI scanning was performed for patients 1–7 days preoperatively in a 3 T MRI scanner (Achieva 3 T, Philips Medical System, Netherlands) for T1‐weighted gradient‐echo sequences with an intravenous contrast‐agent (TR/TE: 9/4 ms, 1 mm^3^ iso‐voxel, Dotagraf 0.5 mmol/ml, by Jenapharm GmbH & Co. KG, Jena, Germany) (T1 + C). MRI data were imported to the nTMS system (version 5.1.1; Nexstim Plc, Helsinki, Finland) to perform navigation during the procedure (Polaris Spectra) according to the standard nTMS protocol (Krieg et al., 2017). The biphasic, 50‐mm‐radius, figure‐8 magnetic stimulation coil was simultaneously tracked to navigate the stimuli.

MEPs were collected as responses to stimuli through electromyography electrodes (Ag/AgCl electrode, Neuroline 720, Ambu) on the right abductor pollicis brevis, abductor digiti minimi, and flexor carpi radialis muscle, which were corrected by a grounding electrode placed on the left flexor carpi radialis muscle (Krieg, 2017; Krieg et al., 2017; Vitikainen et al., 2013). We individually determined the resting motor threshold (rMT) before the mapping procedure, defined as the minimal stimulation intensity at which 50% of the stimuli induced MEPs >50 mA. For the motor mapping, stimuli were output at an intensity of 110% of the individual rMT value. Median nerve‐related MEPs were based on the abductor pollicis brevis muscle and flexor carpi radialis muscle, while ulnar nerve‐related MEPs were based on the abductor digiti minimi muscle. Then, cortical targets with MEPs above 50 mA were regarded as positive stimuli (PS). Three categories of PSs were, respectively, exported as Digital Imaging and Communications in Medicine (DICOM) format in T1 + C space, consisting of median nerve‐related PS (MPS), ulnar nerve‐related PS (UPS), and all PS (APS).

### 2.4 Cortical analysis

PS and T1 + C images from both groups were first co‐registered to the brain template (AAL90) for normalization. Specifically, coordinate axes were defined as x for right‐to‐left, y for inferior‐to‐superior, and z for posterior‐to‐anterior.

Subsequently, coordinates from MPS, UPS, and APS were, respectively, used to calculate CoGs using Euclidean equation, and aspect ratios (ARs) as well as mapping sizes (MSs) in Matlab (Version: 2016b, https://www.mathworks.com/) (Julkunen, 2014; Pitkanen et al., 2018; Sollmann et al., 2020; Zhang et al., 2020): AR is a ratio of lengths of the positive mapping region along the electric field direction and along the perpendicular direction (AR >1: motor regions elongated in the direction of the electric field; AR <1: motor regions elongated the vertical direction of the electric field) (Pitkanen et al., 2018; Sollmann et al., 2020). AR refers to the shape of the mapping area, and MS refers to the area size (Julkunen, 2014; Pitkanen et al., 2018; Sollmann et al., 2020; Zhang et al., 2020). Distributions of mapping regions and CoGs were projected to the AAL90 template to identify their localizing gyrus and displayed as heat maps in the Matlab toolbox BrainNetViewer (Xia et al., 2013).

### 2.5 Subcortical analysis

MPS, UPS, and APS in DICOM format were imported into Brainlab Element (version 3.1.0; Brainlab AG, Munich, Germany) and set as seeds to fuse with T1 + C and DTI files. Then, seed‐based deterministic fiber tracking was performed for their corresponding CST. FA thresholds were set at 0.1 and 0.15, minimal fiber length at 100 mm, and the angulation threshold at 20°. MPS, UPS, and APS were transferred into regions of interest by adding rims of 2 mm to each PS and used for CST tracking with a region of interest of the brain stem. Their CST volume and average FA were calculated and recorded as MPS‐related CST volume (median‐volume) and average FA (median‐FA), UPS‐related CST volume (Ulnar‐volume) and average FA (Ulnar‐FA), and APS‐related CST volume (All‐volume) and average FA (All‐FA).

Additionally, all images were registered to the AAL template to identify whether CoGs were localized within or outside cortical endpoints of the CST.

### 2.6 Statistical analysis

Statistical calculations were performed in GraphPad Prism (version 8.0; GraphPad Software Inc., La Jolla, CA, USA, https://www.graphpad.com/scientific-software/prism/). The level of significance was at *p* < .05. Data distribution was assessed through the Shapiro–Wilk‐Normality test. Accordingly, the independent *t* test or the Mann–Whitney *U* test was used to compare G1 and G2. Descriptive statistics regarding CoG coordinates and tractography characteristics (FA and CST volumes) were analyzed. Results are reported as mean ± standard deviation with ranges. The chi‐squared test or Fisher's exact tests were used to assess mapping regions and CoG locations.

## 3 RESULTS

### 3.1 Demographic analysis

#### 3.1.1 Mean

The average age was 58.0 ± 14.1 years (G1:58.1 ± 16.5 years, G: 58.3 ± 10.8 years; *p* = .982). The rate of metastasis and glioma was, respectively, 19.0 and 81.0% in group G1 and 12.5 and 87.5% in G2 (K‐square test, *p* = .459).

### 3.2 Mapping region analysis

**FIGURE 2:**
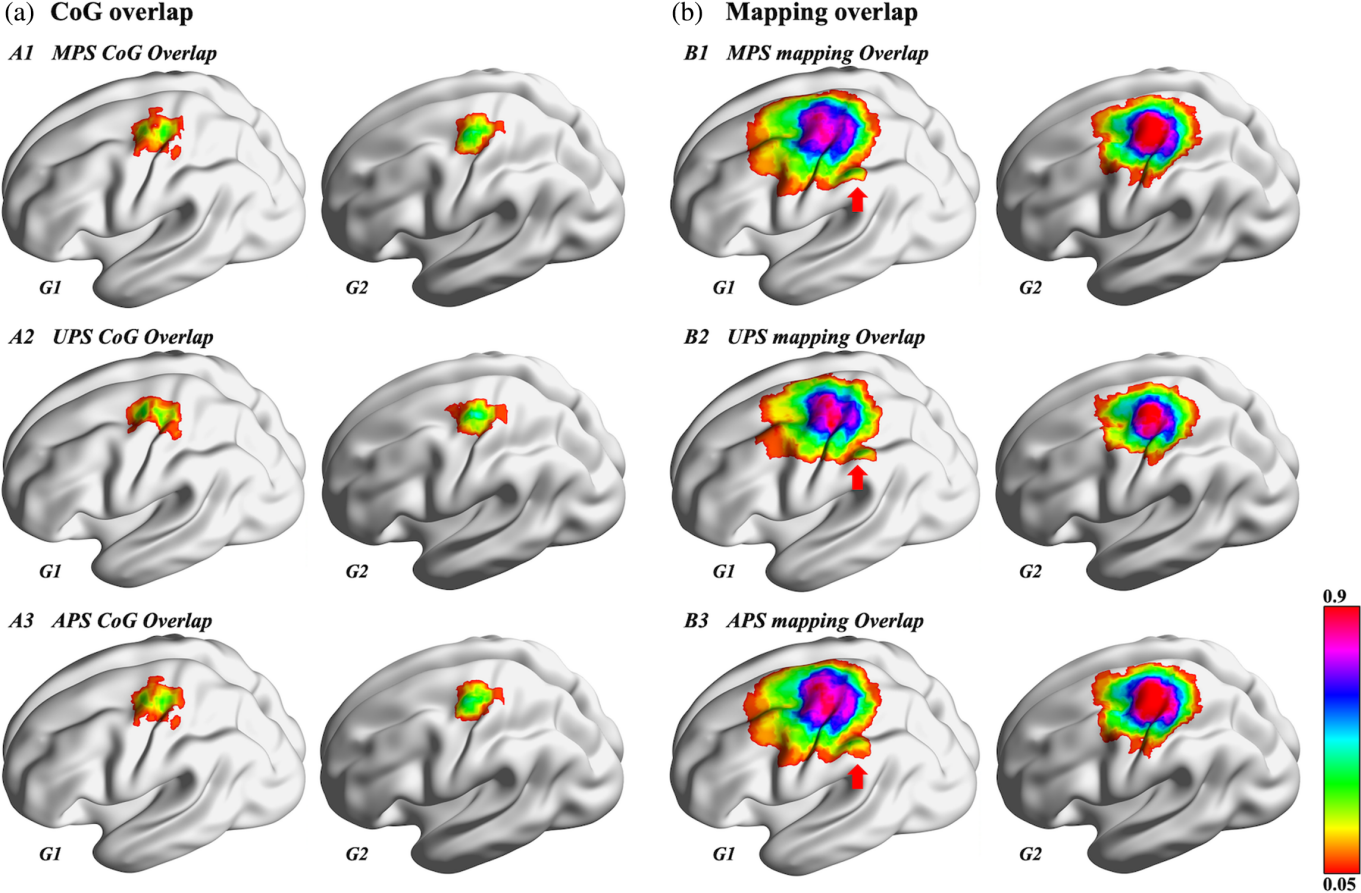
Overlap of center of gravity (CoG) and mapping regions. This figure shows the overlap of CoGs per group (a) and the overlap of mapping regions per group (b) of median nerve‐related positive stimuli (MPS), ulnar nerve‐related positive stimuli (UPS), and all positive stimuli (APS) from the group of patients with tumors inside the left corticospinal tract (CST) or precentral gyrus (G1) and group of patients with tumors outside the left CST or precentral gyrus (G2). The supramarginal gyrus (red arrow) was mapped in patients from G1 but not G2, as seen in B3.

Mapping regions were mainly located in the precentral gyrus, postcentral gyrus, superior parietal gyrus, inferior parietal gyrus, superior frontal gyrus, middle frontal gyrus, and the supplementary motor area in both groups (Table 1). Motor regions in the supramarginal gyrus were found significantly more often in G1 than in G2 patients (*p* = .006; Table 1, Figure 2), as revealed by Fisher's exact test. In G1, muscle strengths (median: Grade 4; range: Grades 1–4+) from patients with the supramarginal gyrus positively mapped did not differ from the muscle strengths in the remaining patients of G1 (median Grade: 4; range: Grades 1–4+) (*p* = .192). The inferior parietal gyrus was more frequently mapped positively in G1, approaching significance levels regarding APS (APS: *p* = .059, Table 1).

**TABLE 1:** Chi‐square analysis on mapping regions.

Gyrus	nTMS	MPS			UPS			APS		
		G1	G2	Results	G1	G2	Results	G1	G2	Results
Precentral	Mapped	21	24		20	24		21	24	
	Not mapped	0	0		0	0		0	0	
Postcentral	Mapped	21	24		19	22	Fisher *p* = 1.000	21	24	
	Not mapped	0	0		2	2		0	0	
Superior parietal	Mapped	11	8	K = 1.666 *p* = .197	11	6	K = 3.572 *p* = .059	12	9	K = 1.736 *p* = .188
	Not mapped	10	16		10	18		9	15	
Inferior parietal	Mapped	9	5	K = 2.535 *p* = .111	9	5	K = 2.535 *p* = .111	11	6	K = 3.572 *p* = .059
	Not mapped	12	19		12	19		10	18	
Superior frontal	Mapped	18	17	K = 1.435 *p* = .231	16	14	K = 1.607 *p* = .205	18	17	K = 1.435 *p* = .231
	Not mapped	3	7		5	10		3	7	
Middle frontal	Mapped	14	15	K = 0.085 *p* = .771	13	12	K = 0.643 *p* = .423	15	15	K = 0.402 *p* = .526
	Not mapped	7	9		8	12		6	9	
Supramarginal	Mapped	10	2	Fisher *p* = .006	10	2	Fisher *p* = .006	10	2	Fisher *p* = .006
	Not mapped	11	22		11	22		11	22	
SMA	Mapped	3	1	Fisher *p* = .326	1	1	Fisher *p* = 1.000	3	2	Fisher *p* = .652
	Not mapped	18	23		20	23		18	22	

*Note*: There are no differences in mapping regions between G1 (patients with tumors within left CST or precentral gyrus) and G2 (patients with tumors outside left CST or precentral gyrus) except the supramarginal gyrus, including regions based on MPS, UPS, and APS (MPS: *p* = .029; UPS: *p* = .029; and APS: *p* = .007).

Abbreviations: APS, all positive stimuli; MPS, median nerve‐related positive stimuli; SMA, supplementary motor area; UPS, ulnar nerve‐related positive stimuli.

The CoGs were found on the precentral, postcentral, and superior frontal gyrus in both groups. The median and ulnar nerve‐related CoGs and the APS CoGs were mainly in the precentral gyrus. Fischer's test showed no significant intragroup differences for CoG locations between MPS, UPS, and APS (G1: *p* = .618; G2: *p* = .830), either no intergroup differences (Supplementary Table 2).

### 3.3 CoG, AR, and MS analysis

The two groups had no statistical differences in CoG coordinates and ARs regarding different neural innervations (all *p* > .05; see Supplementary Table 3). MSs of different nerve‐related cortical motor regions differed among intragroup individuals. Since MSs were not normally distributed, the Mann–Whitney test was performed between the two groups, and no differences were revealed regarding MSs of MPS, UPS, and APS (Supplementary Table 3).

### 3.4 Subcortical analysis

**FIGURE 3:**
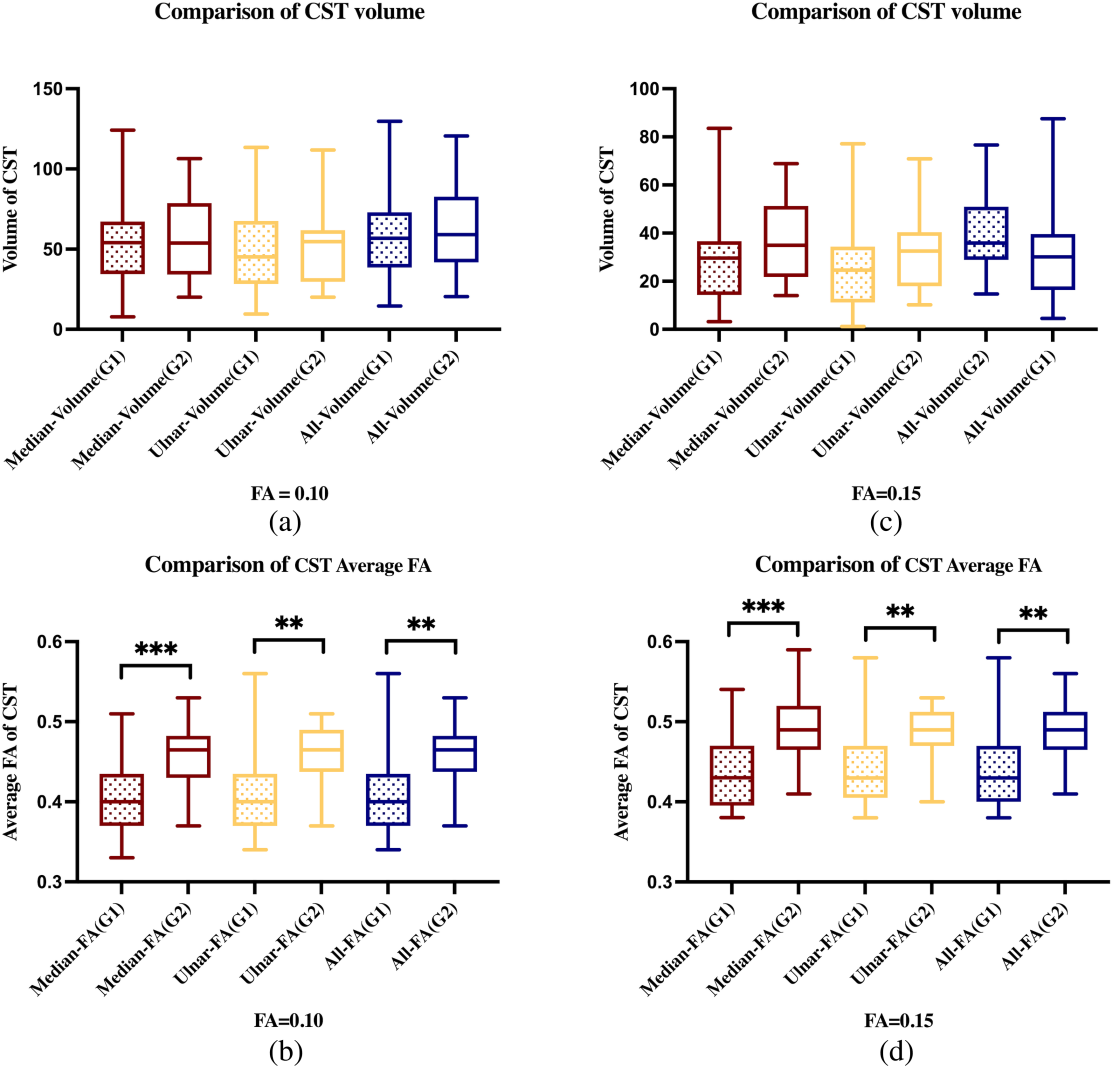
Comparison of corticospinal tract (CST) volumes and average fractional anisotropies (FAs). This figure presents the subcortical comparison of CST volume and average FA between the group of patients with tumors inside the left CST or precentral gyrus (G1, with shading) and the group of patients with tumors outside the left CST or precentral gyrus (G2, without shading). Seeds from the group of patients with tumors inside the left CST or precentral gyrus (G1; 21 cases) and the group of patients with tumors outside the left CST or precentral gyrus (G2; 22 cases) were, respectively, created based on median nerve‐related positive stimuli (MPS, red), ulnar nerve‐related positive stimuli (UPS, yellow), and all positive stimuli (APS, blue). Next, CST fiber tracking thresholding at 0.1 and 0.15 was performed. Their CST volume and average FA were recorded as MPS‐related CST volume (median‐volume) and average FA (median‐FA), UPS‐related CST volume (Ulnar‐volume) and average FA (Ulnar‐FA), and APS‐related CST volume (All‐volume) and average FA (All‐FA). *p < .05; **p < .01; *** *p* < .001.

**FIGURE 4:**
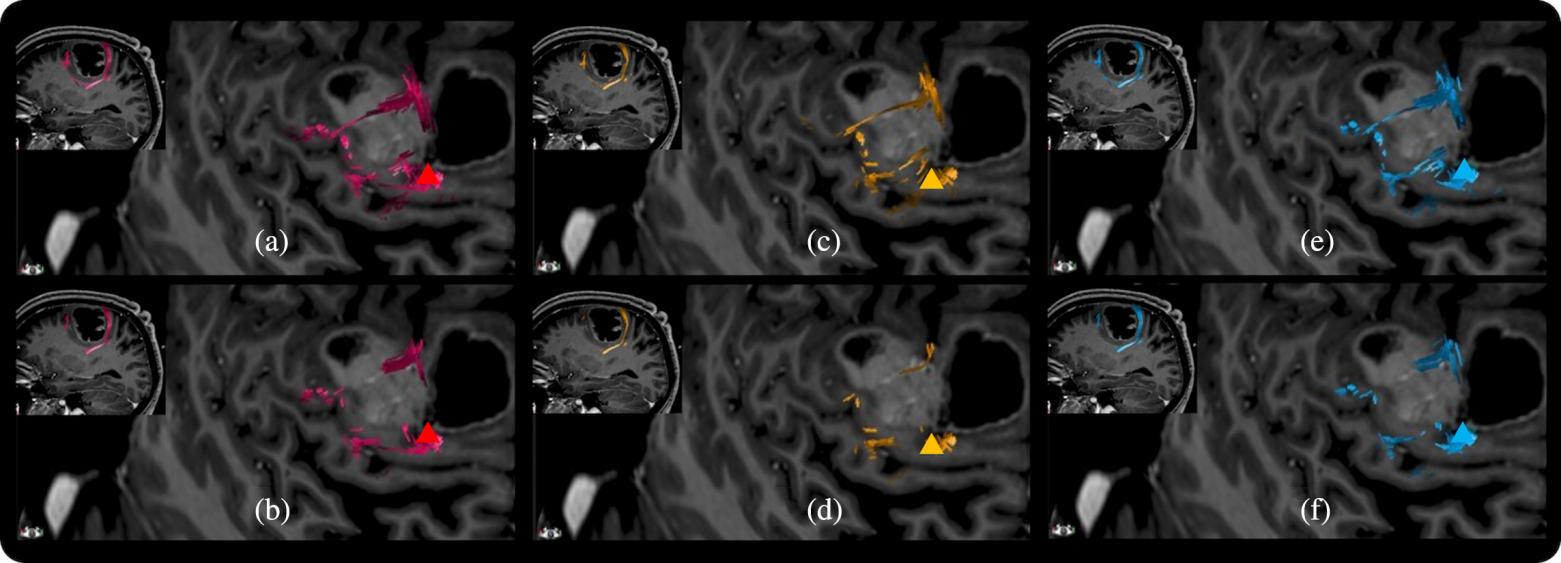
Center of gravity (CoG) and corticospinal tract (CST). This figure illustrates case 4 in group 1. Subfigures a, c, and e represent tractography with fractional anisotropy (FA) thresholding at 0.1, while subfigures b, d, and f are tractography based on FA thresholding at 0.15. Triangles illustrate locations of CoGs, respectively, for median nerve‐related positive stimuli (MPS), ulnar nerve‐related positive stimuli (UPS), and all positive stimuli (APS). Red triangles and fibers are based on MPS (subfigures a and b), yellow is based on UPS (subfigures c and d), and blue is based on APS (subfigures e and f).

Preoperative DTI scans were unavailable for two patients in G2 (Nos. 12 and 16). Fiber tracking was successfully performed in the remaining 22 patients from G2 and all 21 patients from G1. Under both FA thresholds, Median‐Volume (*p* = .654 and *p* = .417), Ulnar‐Volume (*p* = .545, and *p* = .309), and All‐Volume (*p* = .901 and *p* = .417) did not differ between G1 and G2 (Table 2, Figure 3). However, Median‐FA (*p* < .001 and *p* < .001), Ulnar‐FA (*p* = .001, and *p* = .008), and All‐FA (*p* = .002 and *p* = .005) were significantly higher in G2 than in G1 (Table 2, Figure 3). CoGs based on MPS, UPS, and APS were located within the scopes of their corresponding CSTs in both groups (Figure 4).

**TABLE 2:** Comparison of average CST volume and average FA.

Items	G1 (*n* = 21)	G2 (*n* = 22)	*p*‐Value
FA = 0.10			
Median‐volume (cm^3^)	55.298 ± 25.321	58.786 ± 26.294	.654
Ulnar‐volume (cm^3^)	48.012 ± 26.057	51.050 ± 23.635	.545
All‐volume (cm^3^)	58.330 ± 26.005	62.873 ± 26.630	.901
Median‐FA	0.410 ± 0.039	0.458 ± 0.038	<.001
Ulnar‐FA	0.412 ± 0.054	0.459 ± 0.037	.001
All‐FA	0.414 ± 0.056	0.460 ± 0.038	.002
FA = 0.15			
Median‐volume (cm^3^)	27.878 ± 18.770	32.230 ± 16.861	.417
Ulnar‐volume (cm^3^)	26.519 ± 18.892	31.661 ± 14.554	.309
All‐volume (cm^3^)	31.294 ± 19.761	39.653 ± 16.561	.413
Median‐FA	0.441 ± 0.047	0.485 ± 0.040	<.001
Ulnar‐FA	0.445 ± 0.051	0.486 ± 0.036	.008
All‐FA	0.446 ± 0.052	0.486 ± 0.037	.005

*Note*: This table presents CST volume and average FA regarding different seeds, applied for CST fiber tracking thresholding at 0.1 and 0.15 from the group of patients with tumors inside the left CST or precentral gyrus (G1; 21 cases) and the group of patients with tumors outside the left CST or precentral gyrus (G2; 22 cases), including regions based on MPS, UPS, and APS. Their respective average CST volume and average FA were calculated and recorded as median‐volume and median‐FA for MPS‐related CST, Ulnar‐volume and Ulnar‐FA for UPS‐related CST, and All‐volume and All‐FA for APS‐related CST.

Abbreviations: APS, all positive stimuli; CST, corticospinal tract; FA, fractional anisotropy; MPS, median nerve‐related positive stimuli; SMA, supplementary motor area; UPS, ulnar nerve‐related positive stimuli.

## 4 DISCUSSION

The current study investigated the preoperative distribution of motor function based on neural innervations identified by nTMS and their corresponding subcortical structures. We demonstrated that motor regions shifting toward the supramarginal gyrus tended to occur in patients with tumors located within the primary motor regions. However, the locations of CoGs did not significantly differ between both groups. This supports the hypothesis of an “anchor‐and‐ship theory” for motor reorganization processes: motor CoGs are located within or surrounding the CST projecting cortical regions, like a dropped anchor, while the cortical motor regions, as seen in the mapping results, relocated to nearby gyri, like a ship moving around its anchor.

### 4.1 nTMS‐based cortical motor regions

Our analysis revealed that the most significant difference between groups was the presence of positive motor stimuli in the supramarginal gyrus in G1 (eight vs. two patients, Figure 2). Previous literature has mentioned that the supramarginal gyrus is part of the parietal lobe and related to motor reorganizations (Kroliczak et al., 2016; Miao et al., 2018; Wang et al., 2019). Kroliczak et al. found a left‐lateralized extensive increase in activity in the supramarginal gyrus during motor planning (Kroliczak et al., 2016). Miao et al. showed that well‐recovered patients after stroke exhibited significantly increased cerebral blood flows in the contralesional supramarginal gyrus (Miao et al., 2018). Another fMRI study showed that stroke patients receiving motor imagery training presented increased functional connectivity in the supramarginal gyrus and ipsilesional precentral and postcentral gyri (Wang et al., 2019).

The main reason for motor reorganization induced in patients with low‐grade gliomas was attributed to its long disease period in the study by Duffau (2005). However, the current study demonstrated that motor regions lost by fast‐growing malignant tumors can also induce functional redistribution or shifts to adjacent cortical areas. Pathological differences are potential confounding factors, particularly edema. While intracranial metastases result in vasogenic edema, glioma cells can be scattered in peritumoral regions and lead to infiltrative edema. In G1, there were more patients with metastatic tumors (19.1 vs. 12.5%); however, differences in the pathological diagnoses between groups were not statistically significant (*p* = .459). The grouping in this study was based on the location of the tumor entity, specifically on its tissue disruption. It is commonly assumed that the functional impairment caused by GBM would be more severe and that anaplastic astrocytoma and metastatic tumors might be milder. However, despite more gliomas in G2, their preoperative motor functions were intact. Additionally, because all brains were structurally aligned to a standard template, and positively localized regions could be visualized during nTMS mapping, the tumor compression effects on the results can be ruled out. In addition, this article investigates the impact caused by malignant tumors on the functional organization before surgery, and it involves no operation‐related changes. Notably, since it is impossible to map motor regions before tumorigenesis, we cannot infer the extent of loss in the motor areas by tumorigenesis.

### 4.2 Distribution of CoGs, AR, and MS of nTMS motor mapping

In the current study, MSs and ARs did not differ between the two investigated groups. These findings go along with recent literature—Lam et al. found no significant differences in MSs between tumorous and healthy hemispheres (Lam et al., 2019). Moreover, the mapped shape did not change with either stimulation intensity or muscle activation at a fixed stimulating intensity (van de Ruit & Grey, 2016).

The CoG was calculated to reflect the core region of the cortical motor distribution (Kallioniemi et al., 2016; Pitkanen et al., 2018). A study by Koenraadt et al. on TMS motor mapping demonstrated the reproducibility of CoGs based on nTMS, which can be applied as an indicator for motor organization (Koenraadt et al., 2011).

The current study investigated CoGs based on peripheral nerve innervations, which did not differ in location and coordinates between G1 and G2. CoGs in both groups were located in the cortex directly adjacent to the CST, especially in the precentral gyrus (66.7% in G1 and 75.0% in G2), which aligns with findings in previous studies enrolling healthy subjects (Classen et al., 1998). In other words, CoGs were mainly found in the hand knob region and are considered to correspond to the location of the highest density of corticospinal neurons in the precentral gyrus (Yousry et al., 1997). This supports Classen's study showing that mean TMS‐derived CoG projections are near the mean positron emission tomography‐derived CoG in the precentral gyrus (Classen et al., 1998). However, Barz et al. compared preoperatively and postoperatively pooled CoGs from stimuli related to the abductor pollicis brevis muscle in glioma patients and reported significantly different locations compared to healthy individuals (Barz et al., 2018). Nevertheless, their article did not mention the site in the gyri hosting the shifted CoGs, which does not conflict with this study's conclusion that CoGs majorly remain in the precentral gyrus. We suggest that the following two aspects are potentially essential to interpret this study's results: (1) the current study included no healthy controls. All enrolled patients for G1 and G2 suffered from malignant tumors, usually with a shorter disease period until diagnosis and (2) individual motor reorganization may lead to CoG shifts, but it is suggested that they remain within CST cortical endpoints. A systematic review in 2020 identified the cortical motor reorganization in the affected hemisphere following an amputation and showed CoG relocation majorly in the precentral gyrus (Gunduz et al., 2020). Therefore, these findings suggest that particular care needs to be taken to preserve the CoG site and its surrounding brain structures in the precentral gyrus when removing cerebral tumors, which is essential for the output of motor function.

### 4.3 Subcortical analysis

Under both FA thresholds, no difference was detected between groups for different innervation‐based CST volumes. There are two ways to interpret this result: (1) the impact of gliomas affects both the local and distant microenvironment and signaling, as demonstrated by previous studies (Gao et al., 2020; Mandal et al., 2020), but whether tumors outside motor regions have an impact on CSTs has not been clarified according to our knowledge and (2) the CST reconstruction process is simultaneously affected by multiple factors, which results in the insensitivity of its volumes to the tumors' impacts. In a study by Reich et al., reconstructed CST fiber counts and tract volumes were highly variable, rendering themselves insensitive to abnormalities in diseases (Reich et al., 2006). Given the limitations of the existing imaging techniques, this study cannot yet support the first explanation, and the second explanation is favored.

Regarding comparisons of average FA values of the CST corresponding to different innervations, statistical significances between G1 and G2 were present for both FA thresholds. Previous studies suggest that FA is related to motor alterations and functional plasticity (Imfeld et al., 2009; Yu et al., 2007). Yu et al. demonstrated that significantly increased average FA of the CST was found in early blind men but not in early blind women, which was attributed to the changes in motor experience during the critical development of the CST (Yu et al., 2007). An investigation in musicians showed that training‐induced changes in the axonal membranes of the CST might lead to increased radial diffusivity, as reflected in decreased FA values (Imfeld et al., 2009). Therefore, it can be concluded that a decreased FA of the CST might be related to increased functional plasticity.

Second, from a histopathological point of view, FA was also significantly correlated with axonal loss in the CST in tumor patients (Fekonja et al., 2020; Min et al., 2017). Alterations of FA of the CST were correlated with the Fugl‐Meyer assessment in stroke rehabilitation and were found to be a reliable prognosis indicator (Doughty et al., 2016; Zolkefley et al., 2021). Hence, from the lower average FA detected in G1 patients, we can confirm that their motor structures are genuinely affected by tumor growth. It remains unclear whether the decrease in FA of the CST is a pathological manifestation caused by glioma or an indicator of structural reorganization or plasticity in response to impaired function.

### 4.4 Potential clinical applications of the theory

First, the proposed “anchor‐and‐ship theory” enhances the preoperative planning. It helps to identify regions essential for motor recovery and guides neurosurgeons to protect these areas. Second, with a clearer understanding of motor structural reorganization, rehabilitation strategies can be tailored more effectively to intervene in specific regions to induce compensation of motor function. Third, our findings can be applied in radiotherapy planning, as the avoidance of critical cortical and subcortical motor areas decreases the risk of radiation‐induced motor deficits. Finally, the theory provides further insights into research on neural plasticity, especially in pathological conditions, such as tumor invasion.

Overall, the findings and theory proposed in the current study hold promise for improving clinical outcomes and the understanding of neuro‐oncology.

### 4.5 Study limitations

The limitations of this study should be considered when interpreting the results. First, the cohort used in this study was heterogeneous, including various glioma subtypes and metastases, without further subgroup differentiation. This heterogeneity might have introduced variability in the results.

Our data primarily relied on preoperative mapping, lacking long‐term follow‐up data concerning postoperative motor distribution. Therefore, our understanding of the dynamic changes in motor function over time remains limited.

Finally, we did not have access to data on the distribution of motor function in the contralateral hemisphere or in a healthy control cohort. This absence of comparative data made it challenging to assess the full extent of motor function changes solely based on our patient cohort. Future studies with more specific patient subgroups and comprehensive long‐term data are warranted to address these limitations and provide a more comprehensive understanding of motor function in brain tumor patients.

## 5 CONCLUSION

Functional deficits induced by fast‐growing malignant tumors can still involve motor function reorganization at cortical and subcortical levels when invading motor regions. Based on the current study, the hypothesis of an “anchor‐and‐ship theory” for motor reorganization, which can benefit from anticipating functional reorganization and can be used as a neuro‐oncological tool for local therapy, like surgery or radiotherapy, was proposed.

## FUNDING INFORMATION

This research received no specific grant from funding agencies in the public, commercial, or not‐for‐profit sectors.

## CONFLICT OF INTEREST STATEMENT

BM received honoraria, consulting fees, and research grants from Medtronic (Meerbusch, Germany), Icotec AG (Altstätten, Switzerland), Nexstim Plc (Helsinki, Finland), and Relievant Medsystems Inc. (Sunnyvale, CA, USA), honoraria, and research grants from Ulrich Medical (Ulm, Germany), honoraria and consulting fees from Spineart Deutschland GmbH (Frankfurt, Germany) and DePuy Synthes (West Chester, PA, USA), and royalties from Spineart Deutschland GmbH (Frankfurt, Germany). SMK is a consultant for Ulrich Medical (Ulm, Germany) and Need Inc. (Santa Monica, CA, USA) and received honoraria from Nexstim Plc (Helsinki, Finland), Spineart Deutschland GmbH (Frankfurt, Germany), Medtronic (Meerbusch, Germany) and Carl Zeiss Meditec (Oberkochen, Germany). SMK and BM received research grants and are consultants for Brainlab AG (Munich, Germany). SI is a consultant for Brainlab AG (Munich, Germany) and received honoraria and consulting fees from Icotec AG (Altstätten, Switzerland), Spineart Deutschland GmbH (Frankfurt, Germany), Carl Zeiss Meditec (Oberkochen, Germany), and Nexstim Plc (Helsinki, Finland). All authors declare no conflict of interest regarding the materials used and the results presented in this study. The funders had no role in the study's design, in the collection, analysis, or interpretation of data, the writing of the manuscript, or the decision to publish the results.

## Supporting information


**DATA S1** Supporting Information.

## Data Availability

In the interest of patient privacy, all the collected raw data of individual cases is imparticipable. The anonymous datasets used and analyzed during the current study, the study protocol, and the statistical analysis plan are available upon reasonable request from the corresponding author to researchers who provide a methodologically sound proposal. Proposals should be directed to sebastian.ille@tum.de; data requestors will be required to sign a data access agreement to gain access.
