# Supplementary material for: Motor mapping of the hand muscles using peripheral innervation‐based navigated transcranial magnetic stimulation to identify functional reorganization of primary motor regions in malignant tumors
Source: Hum Brain Mapp. 2024 Mar 4;45(4):e26642. doi: 10.1002/hbm.26642 (PMC10910269; doi:10.1002/hbm.26642)
Supplement: Supplementary file 1 — DATA S1 Supporting Information. [file HBM-45-e26642-s001.docx]

**Supplementary Table 1. Demographic data**

| No. | Gender | Age (Year) | Location | Muscle Strengths | Pathological diagnosis |
| --- | --- | --- | --- | --- | --- |
| G1 | | | | | |
| 1 | Female | 56 | Precentral | 4/5 | Metastasis |
| 2 | Male | 52 | Frontal | 4/5 | Glioblastoma, WHO CNS 4, IDH- Wildtype |
| 3 | Female | 61 | Frontal | 2/5 | Glioblastoma, WHO CNS 4, IDH- Wildtype |
| 4 | Male | 47 | Precentral | 1/5 | Glioblastoma, WHO CNS 4, IDH- Wildtype |
| 5 | Male | 59 | Frontal | 5-/5 | Glioblastoma, WHO CNS 4, IDH- Wildtype |
| 6 | Male | 53 | Precentral, Parietal | 5-/5 | Metastasis |
| 7 | Male | 72 | Temporal | 1/5 | Glioblastoma, WHO CNS 4, IDH- Wildtype |
| 8 | Female | 76 | Frontal, Precentral | 4/5 | Glioblastoma, WHO CNS 4, IDH- Wildtype |
| 9 | Female | 31 | Precentral | 4/5 | Anaplastic Astrocytoma, WHO CNS 3, IDH- Wildtype |
| 10 | Male | 44 | Thalamus | 4/5 | Glioblastoma, WHO CNS 4, IDH- Wildtype |
| 11 | Male | 53 | Frontal | 3/5 | Glioblastoma, WHO CNS 4, IDH- Wildtype |
| 12 | Male | 66 | Postcentral | 4/5 | Glioblastoma, WHO CNS 4, IDH- Wildtype |
| 13 | Male | 70 | Frontal | 4/5 | Glioblastoma, WHO CNS 4, IDH- Wildtype |
| 14 | Male | 73 | Parietal | 4/5 | Glioblastoma, WHO CNS 4, IDH- Wildtype |
| 15 | Female | 49 | Insula | 5-/5 | Anaplastic Astrocytoma, WHO CNS 3, IDH-Mutant |
| 16 | Female | 50 | Insula | 5-/5 | Anaplastic Astrocytoma, WHO CNS 3, IDH-Mutant |
| 17 | Male | 32 | Frontal | 2/5 | Anaplastic Astrocytoma, WHO CNS 3, IDH-Mutant |
| 18 | Female | 81 | Precentral | 5-/5 | Glioblastoma, WHO CNS 4, IDH- Wildtype |
| 19 | Male | 74 | Precentral, Frontal | 4/5 | Metastasis |
| 20 | Female | 68 | Precentral | 1/5 | Glioblastoma, WHO CNS 4, IDH- Wildtype |
| 21 | Male | 73 | Frontal | 4/5 | Metastasis |
| G2 | | | | | |
| 1 | Male | 61 | Frontal | 5/5 | Metastasis |
| 2 | Male | 75 | Temporal | 5/5 | GBM, WHO CNS 4, IDH-Wildtype |
| 3 | Male | 33 | Frontal | 5/5 | Oligodendroglioma, WHO CNS 3, IDH-Mutant |
| 4 | Male | 48 | Frontal | 5/5 | GBM, WHO CNS 4, IDH-Mutant |
| 5 | Male | 57 | Frontal | 5/5 | Anaplastic Astrocytoma, WHO CNS 3, IDH-Mutant |
| 6 | Male | 78 | Temporal | 5/5 | GBM, WHO CNS 4, IDH-Wildtype |
| 7 | Male | 55 | Temporal | 5/5 | GBM, WHO CNS 4, IDH-Wildtype |
| 8 | Male | 62 | Temporal | 5/5 | GBM, WHO CNS 4, IDH-Wildtype |
| 9 | Male | 65 | Temporal | 5/5 | GBM, WHO CNS 4, IDH-Wildtype |
| 10 | Male | 51 | Frontotemporal | 5/5 | Anaplastic Astrocytoma, WHO CNS 3, IDH-Wildtype |
| 11 | Male | 62 | Parietooccipital | 5/5 | GBM, WHO CNS 4, IDH-Wildtype |
| 12 | Male | 45 | Temporal, Frontal | 5/5 | Anaplastic Astrocytoma, WHO CNS 3, IDH-Mutant |
| 13 | Male | 50 | Temporal | 5/5 | Astrocytoma, WHO CNS 3, IDH-Wildtype |
| 14 | Male | 68 | Frontal | 5/5 | GBM, WHO CNS 4, IDH-Wildtype |
| 15 | Male | 54 | Temporal | 5/5 | GBM, WHO CNS 4, IDH-Wildtype |
| 16 | Male | 68 | Parietooccipital | 5/5 | GBM, WHO CNS 4, IDH-Wildtype |
| 17 | Male | 47 | Frontal | 5/5 | GBM, WHO CNS 4, IDH-Wildtype |
| 18 | Female | 74 | Frontal | 5/5 | Metastasis |
| 19 | Male | 66 | Parietal | 5/5 | GBM, WHO CNS 4 |
| 20 | Female | 57 | Parietooccipital | 5/5 | Anaplastic Astrocytoma, WHO CNS 3 |
| 21 | Male | 34 | Frontal | 5/5 | Anaplastic Astrocytoma, WHO CNS 3 |
| 22 | Female | 72 | Temporal | 5/5 | Metastasis |
| 23 | Male | 58 | Parietooccipital | 5/5 | GBM, WHO CNS 4, IDH-Wildtype |
| 24 | Female | 52 | Frontal | 5/5 | GBM, WHO CNS 4, IDH-Mutant |

*This table presents demographic data of all patients enrolled in the current study. Pre-operative scores for muscle strength were collected in both groups. G1 patients with tumors within motor regions presented motor deficits at variable levels. G2 patients showed full muscle strength because CST-related cortical and subcortical regions were not affected. All pathological diagnoses were confirmed postoperatively according to the World Health Organization Classification of tumors of the central nervous system. In G2, DTI data was not available for patients 12 and 16.*

**Supplementary Table 2. Comparison of CoG location**

| Gyrus | G1 | | | G2 | | |
| --- | --- | --- | --- | --- | --- | --- |
|  | **MPS** | **UPS** | **APS** | **MPS** | **UPS** | **APS** |
| Precentral | 13 | 14 | 16 | 18 | 17 | 18 |
| Postcentral | 8 | 6 | 5 | 4 | 4 | 4 |
| Superior frontal | 0 | 1 | 0 | 2 | 3 | 2 |

*This table presents the locations of Centers of Gravity (CoGs) of Group1 (Patients with tumors inside the precentral gyrus or CST; G1) and Group2 ((Patients with tumors outside the precentral gyrus or CST; G2). CoGs of both groups were located in three regions, consisting of the precentral gyrus, postcentral gyrus, and superior frontal gyrus. The non-parametric (Fischer’s Test）analysis detected no differences between the two groups for CoG locations of the median nerve (p=0.108), CoG locations of the ulnar nerve (p=0.577), and general CoG locations ( p=0.547).*

**Supplementary Table 3. Comparison of CoG coordinates and aspect ratio**

| Items | | | G1 | G2 | p-value |
| --- | --- | --- | --- | --- | --- |
| MPS | **CoG**  **(mm)** | **X-axis** | 132.364 ± 8.919 | 132.031 ± 5.377 | 0.903 |
|  |  | **Y-axis** | 193.821 ± 12.478 | 200.869 ± 12.028 | 0.061 |
|  |  | **Z-axis** | 98.633± 9.484 | 102.021 ± 10.103 | 0.255 |
|  | **AR** | | 1.273 ± 0.242 | 1.241 ± 0.396 | 0.744 |
|  | **MS (cm^2^)** | | 5.825 ± 3.874 | 4.162± 2.052 | 0.074 |
| UPS | **CoG**  **(mm)** | **X-axis** | 131.702 ± 9.191 | 131.982 ± 5.956 | 0.903 |
|  |  | **Y-axis** | 194.293 ± 12.288 | 200.985 ± 11.866 | 0.070 |
|  |  | **Z-axis** | 98.564± 10.062 | 101.409 ±10.367 | 0.357 |
|  | **AR** | | 1.269 ± 0.321 | 1.308 ± 0.371 | 0.747 |
|  | **MS (cm^2^)** | | 3.438 ± 2.891 | 2.331 ± 1.407 | 0.103 |
| TPS | **CoG**  **(mm)** | **X-axis** | 132.406 ± 8.963 | 132.083 ± 5.443 | 0.377 |
|  |  | **Y-axis** | 193.576 ± 12.383 | 200.874 ± 11.941 | 0.693 |
|  |  | **Z-axis** | 99.021 ± 9.385 | 101.681 ± 9.780 | 0.359 |
|  | **AR** | | 1.303 ± 0.241 | 1.212 ± 0.371 | 0.060 |
|  | **MS (cm^2^)** | | 6.507 ± 4.097 | 4.691± 1.894 | 0.201 |

*This table presents Centers of Gravity (CoGs) coordinates, aspect ratio (AR), and mapping size (MS) regarding median nerve and ulnar nerve-related positive mapping sites (MPS, and UPS) and all positive mapping sites (APS). AR is a ratio of lengths of the positive mapping region along the electric field direction and the perpendicular direction. The Mann-Whitney test was applied to MS, and the independent T-test was applied to CoG and MS measures for comparisons between G1 and G2. There were no differences in CoG, AR, and MS between Group1 (Patients with tumors inside the precentral gyrus or CST; G1) and Group2 (Patients with tumors outside the precentral gyrus or CST; G2).*
